# Dentin Growth after Direct Pulp Capping with the Different Fractions of Plasma Rich in Growth Factors (PRGF) vs. MTA: Experimental Study in Animal Model

**DOI:** 10.3390/jcm10153432

**Published:** 2021-07-31

**Authors:** José F. Gaviño-Orduña, Javier Caviedes-Bucheli, María C. Manzanares-Céspedes, Sophie Román-Richon, Benjamín Martin-Biedma, Juan J. Segura-Egea, Esther Berástegui-Jimeno, José López-López

**Affiliations:** 1 Department of Odonto-Stomatology, Faculty of Medicine and Health Sciences, School of Dentistry, University of Barcelona, 08907 Barcelona, Spain; sophie.roman@ymail.com (S.R.-R.); eberastegui@gmail.com (E.B.-J.); 2 Centro de Investigaciones Odontologicas (CIO), Pontificia Universidad Javeriana, Bogota 11001000, Colombia; javiercaviedes@gmail.com; 3 Human Anatomy and Embryology Unit, Department of Pathology and Experimental Therapeutics, Faculty of Medicine and Health Sciences, School of Dentistry, University of Barcelona, 08907 Barcelona, Spain; mcmanzanares@ub.edu; 4 Unit of Dental Pathology and Therapeutics II, School of Medicine and Dentistry, University of Santiago de Compostela, 15782 Santiago de Compostela, Spain; b.martinbiedma@gmail.com; 5 Department of Stomatology, School of Dentistry, University of Sevilla, 41009 Sevilla, Spain; segurajj@us.es; 6 Service of the Surgical Medical Area, Odontological Hospital University of Barcelona, University of Barcelona, 08907 Barcelona, Spain; 7 Oral Health and Masticatory System Group—IDIBELL (Bellvitge Biomedical Research Institute), University of Barcelona, 08907 Barcelona, Spain

**Keywords:** pulp capping, regenerative endodontics, pulpotomy, plasma rich growth factors, fluorescence microscopy, vital pulp therapy

## Abstract

Background: This study aimed to evaluate the area of dentin growth in rabbit incisors after pulp capping with plasma rich in growth factors (PRGF) compared with mineral trioxide aggregate (MTA) by fluorescence. Methods: twenty-seven upper and lower incisors of rabbits were divided into 4 groups: poor PRGF (F1) (*n* = 9 teeth), rich PRGF (F2) (*n* = 8 teeth), ProRoot MTA (positive control, *n* = 5 teeth), and untreated (NC) (negative control, *n* = 5). Fluorochrome markers were injected 24 h before surgery and the day before euthanasia, 28 days after the vital pulp therapy (VPT). Two transverse cuts were performed to every tooth: the first cut (A), 1 mm incisal to the gingival margin, and the second cut (B), 5 mm apical to the first cut. The sections were assessed with histomorphometric evaluation by fluorescence microscopy, comparing the dentin area between fluorescence marks and the total mineralized area. Results: The higher percentage of dentin growth was observed in the F2 group (B = 63.25%, A = 36.52%), followed by F1 (B = 57.63%, A = 30,12%) and MTA (B = 38.64%, A = 15.74%). The group with lowest percentage of dentin growth was the NC group (B = 29.22%, A = 7.82%). Significant difference (*p* < 0.05) was found between F2 group and MTA, also statistically significant difference has been observed comparing dentin growth areas of NC group with F1 and F2 groups. Conclusions: The application of PRGF rich and poor fraction as a pulp capping material stimulated dentin formation more intensively than MTA and NC.

## 1. Introduction

Vital pulp therapy (VPT) has been used for the treatment of deep caries and reversible pulpitis with good results, having reported successful outcomes for VPT even in cariously exposed pulps of vital teeth with irreversible pulpitis [1]. VPT is a conservative and restorative dental procedure which prevents the complete removal or excavation of the healthy pulp tissue in teeth with compromised dental pulp [2]. The procedures considered as VPT are indirect or direct pulp capping and partial or full pulpotomy. Traditionally these treatments have been assessed as highly successful in deciduous dentition but reports about their outcome in permanent dentition were inconsistent [2,3].

The success rates of VPT have increased, even in permanent teeth, with an outcome rate of 73.2% for direct pulp capping, 96.4% for partial pulpotomy, and 77.8% for full pulpotomy [1]. These results have been possible thanks to a better understanding of pulp physiology (in the initial stages, a partial irreversible pulpitis could be limited only to the coronal area of the pulp), a proper diagnosis coupled with a careful case selection, improved clinical protocols, the application of bioactive molecules, and new capping materials and the completion of a final restoration. Considering all these concepts, vital pulp therapies have become reliable treatment options for permanent teeth diagnosed with normal pulps, reversible or even initial stages of irreversible pulpitis limited to the coronal area of the pulp [1].

The future of VPT is centered on three points: an improvement of pulpal status diagnosis using biomarkers, the development of capping biomaterials such as bioceramics, and more advancements on the regeneration of the pulp–dentin complex [4,5,6]. Pulp–dentin complex regeneration studies are based on the three pillars for tissue engineering: the source of stem cells (genesis), the supply of growth factors (induction), and the existence of a scaffold (conduction) [4].

Growth factors, like BMP-7, TGF-β1, and VEGF, are responsible for growth by way of the vascularization and mineralization induction during reparation and regeneration of pulp–dentin complex. These bioactive molecules can have a positive effect in direct and indirect pulp capping, improving tertiary dentin formation with less initial inflammatory response, in animal models [7].

High cost is one of the problems for the application of bioactive molecules in VPT. Autologous growth factors offer a cheaper, easier, and more conservative alternative to obtain the same biological responses, which is worth investigating. Plasma rich in growth factors (PRGF) is an autologous scaffold that is obtained by platelet activation and fibrinogen polymerization. Several studies have shown the capacity of this preparation to stimulate collagen production, angiogenesis and cell differentiation, and anti-inflammatory and antibacterial properties have also been reported for PRGF [8,9,10].

Okada et al. (2016) proved that PRGF induces matrix mineralization, accelerating its formation, and their work showed that TGF-β, PDGF-αβ, PDGF-ββ, and VEGF concentrations in supernatant were about 50% less than in rich fraction of PRGF [11].

Due to the small number of studies evaluating the biological effects and the mineralization capacity of PRGF in the pulp–dentin complex, the aim of the present study was to evaluate the difference in tertiary dentin formation on rabbits’ incisors exposed pulp, comparing three different pulp capping materials: poor and rich fraction of PRGF and mineral trioxide aggregate (MTA).

## 2. Materials and Methods

### 2.1. Study Design

To evaluate the dentin growth on rabbits’ incisors exposed pulp after the application of two different fractions of PRGF, a prospective experimental study in animal model, was designed. Eight New Zealand male one-year-old rabbits were submitted to the procedure. Four incisors (two upper and two lower) from each animal (32 incisors) were included in the study. Teeth with any pathology (i.e., local inflammation or necrosis), or those in which hemostasis could not be achieved after pulp exposure, were excluded.

All animals were treated according to the guidelines for the care and use of experimental animals of the European Communities Council Directive of 24 November 1986 (86/609/EEC), complying with Basel Declaration, the ARRIVE guidelines and in accordance with local laws and regulations. Ethical approval for the research was obtained from the Comitè Ètic d’Experimentació Animal de la Universitat de Barcelona (CEEA-UB) number 92/15.

### 2.2. Preparation of PRGF

**Figure 1:**
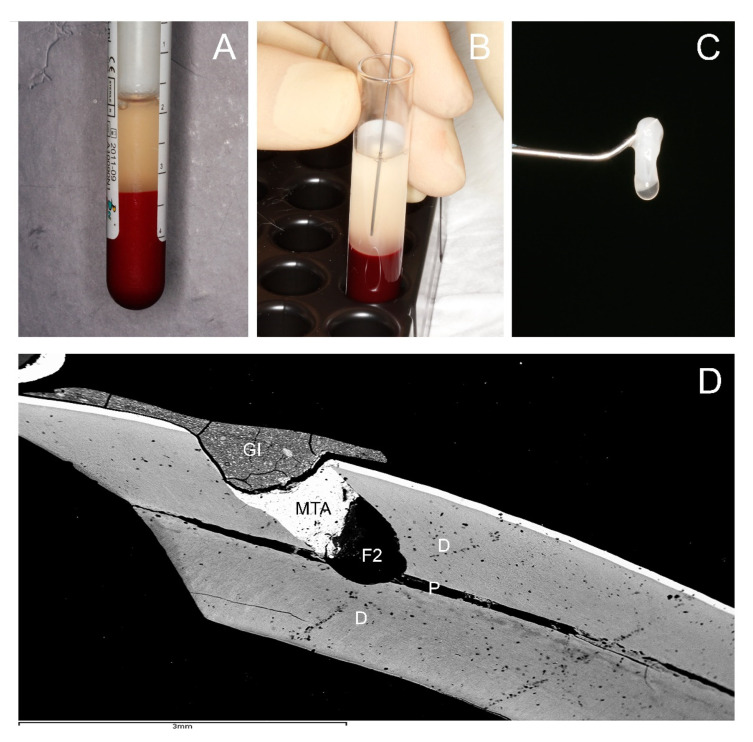
Obtention of the tridimensional clot of PRGF and a rabbit mandibular incisor with the intervention area. (**A**) Detailed view of the extraction tube after centrifugation at 580 mg for 8 min at room temperature using centrifuge system (BTI Biotechnology Institute SL, Vitoria, Spain). (**B**) Detailed view of pipetting technique. (**C**) Detailed view of the activated F2 clot. (**D**) Backscattered scanning electron microscopy (BS-SEM) image ×20, micrograph showing overall morphology of a rabbit mandibular incisor with the intervention area. P: pulp. D: dentin. F2: filling with 2 mm^2^ clot of F2 in contact with the pulp tissue. MTA: covering of 1 mm of MTA (ProRoot mineral trioxide aggregate (MTA); Dentsply Tulsa Dental Specialty, Tulsa, OK, USA). GI: light-curing glass ionomer Vitrebond Plus (3M ESPE, St. Paul, MN, USA), as a final sealing layer.

To not exceed the blood volume limit, 5 mL blood in tubes with 3.8% sodium citrate as an anticoagulant were extracted from each rabbit. Tubes were centrifuged at 580 mg for 8 min at room temperature, using a centrifuge system patented by BTI (BTI Biotechnology Institute SL, Vitoria, Spain) and following the protocol described by described by the manufacturer [12]. The plasma column was afterwards divided into two fractions: fraction 2 (F2) or rich fraction or Plasma rich in growth factor (PRGF), defined as the 1 mL of plasma above the buffy coat, and the fraction 1 (F1) or poor fraction or Plasma poor in growth factor (PPGF), defined as the plasma column above F2. This gave a total of 1 mL of F2 and the F1 volume depending on the hematocrit value of each rabbit (Figure 1A,B). F2 was poured gently into a sterile glass container and activated by adding 50 µL of 10% calcium chloride solution; the latter caused the formation of a tridimensional clot and release of growth factors and proteins of autologous platelets (Figure 1C). A fibrin elastic membrane was obtained maintaining F1 at 37 °C.

### 2.3. Preparation of Rabbit Incisors and Implantation of PRGF

In vivo markers were used to evaluate the mineralization process: 15 mg/kg tetracycline was injected to the rabbits, 24 h before the intervention, and 15 mg/kg of calcein the day before euthanasia, which was performed 28 days after the intervention. These markers were used afterwards to assess dentin growth by means of fluorescence microscopy [13].

The animals were sedated with a subcutaneous injection of Xylazine HCl 5 mg/kg, followed by another of Ketamine HCL 35 mg/kg. A vestibular cavity on the incisors was then made with a contra-angle handpiece (W & H, Bürmoos, Austria), using high-speed (30,000 rev/min) with a 0.8 mm carbide tungsten bur (Komet Dental, Lemgo, Germany) and profuse irrigation with sterile physiological saline. This cavity was performed 1 mm incisal to the marginal gingiva, with an extension of 2 mm^2^ and depth necessary to access to the pulp chamber, with a 45 degrees inclination towards apical. Every four cavities a new bur was used. Pulp exposure was realized with a sterile probe to create minimal bleeding, the wound was rinsed with an antiseptic solution 2.5% NaOCl, and hemostasis was achieved by pressure with cotton balls. If a good hemostasis was not accomplished from 1–2 min up to 10 min, the incisor was excluded from the study.

From each rabbit, four incisors were treated: The teeth were divided into four groups, and the selection of the tooth for every treatment was made randomly: five incisors for negative control group (NC) consisted of untreated teeth, five incisors for positive control group, which only the capping material Mineral trioxide aggregate was placed (ProRoot mineral trioxide aggregate (MTA); Dentsply Tulsa Dental Specialty, Tulsa, OK, USA). The other twenty-two incisors were filled with F1 or F2.

For the F1- and F2-treated teeth, the filling was performed depositing 2 mm^3^ clot of F2 or F1 inside the cavity, covered with 1 mm of MTA, following the manufacturer’s instructions. Light-curing glass ionomer Vitrebond Plus (3M ESPE, St. Paul, MN, USA) was placed as a final sealing layer (Figure 1D). For the control group, the filling was performed placing only MTA on the pulp exposure, covered with light-curing glass ionomer Vitrebond Plus, placed as a final sealing layer.

After the intervention, the animals were administered 0.5 mL Meloxicam (5 mg/mL concentrate), every 24 h for three days, to control postoperative analgesia. Neither behavior alterations nor weight loss were observed in the animals during the postoperative period.

The euthanasia was performed the 28th day after the procedure, by intravenous overdose of thiopental sodium (160 mg/kg). The samples were obtained carefully by dissecting the soft tissues using a scalpel and fracturing the maxilla and mandible with forceps 1 cm apical to the root; the samples were then placed in labeled containers with 10% neutral buffered formalin solution.

### 2.4. Preparation of Samples and Fluorescence

**Figure 2:**
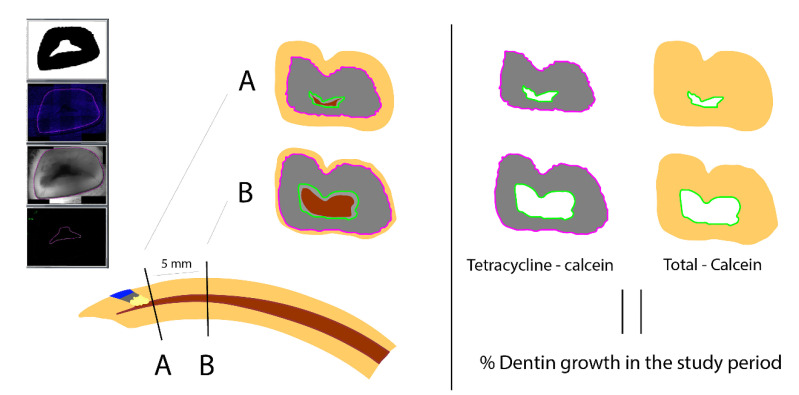
Illustration of the workflow of the sections’ obtention and measurements of dentinogenesis area. Two transverse cuts for each tooth: cut (**A**) 1 mm incisal to the gingival margin and cut (**B**) 5 mm apical to the first cut. Both cuts perpendicular to the curvature of the tooth. Red area: Pulp. Green line: Calcein mark. Purple line: Tetracycline mark. Gray area: Tetracycline mark–Calcein mark. Yellow area: Total mineralized area–Calcein mark. Measurements of different areas and calculation of dentin growth percentage, comparing the area between the 2 fluorescence marks (dentin growth during study time) with respect to the global area of the mineralized tooth excluding the area inside the calcein mark (total mineralized area without dentin growth after calcein application) (using the free download software ImageJ).

To analyze the specimens by fluorescence, the samples were embedded in Technovit 7200 VLC (KULZER GmbH, Hanau, Germany) [14]. To facilitate the analysis, two transverse cuts were performed to the teeth: the first cut (A) at the same level where the perforation had been done, 1 mm incisal to the gingival margin, and the second cut (B), 5 mm apical to the first cut. Both cuts were done perpendicular to the curvature of the tooth (Figure 2).

**Figure 3:**
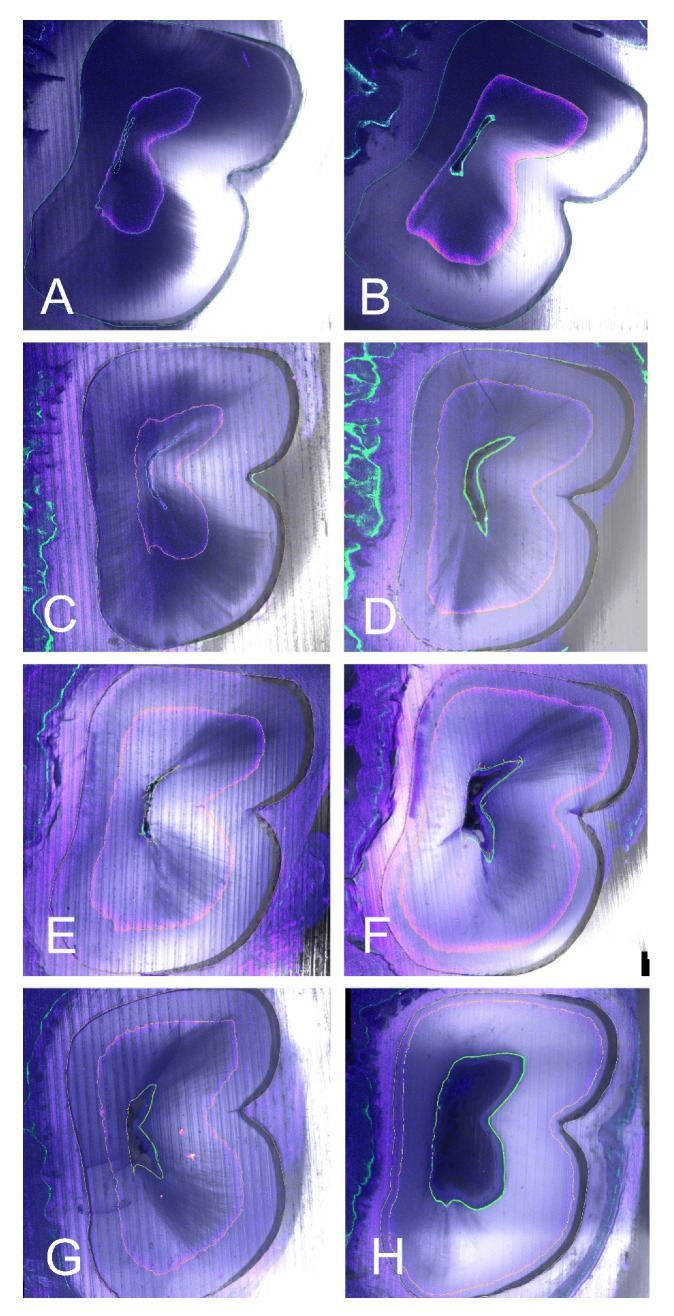
Representative examples of fluorescence images (Leica DM 6000B. Leica Microsystems, Heerbrugg, Switzerland. Leica CTR 6000, Leica Microsys-tems, Heerbrugg, Switzerland), of A sections (**A**,**C**,**E**,**G**) and B sections (**B**,**D**,**F**,**H**). Special filters were used (Ca: calcein green, Te: tetracycline purple). Four different teeth with the different treatments: (**A**,**B**) Negative control. (**C**,**D**) MTA group. (**E**,**F**) poor PRGF. (**G**,**H**) rich PRGF. Note the higher area of dentin growth in rich PRGF group (**G**,**H**) for both A and B sections, followed by poor PRGF group (**E**,F) and MTA group (**C**,**D**). Finally, the lowest dentin growth area for NC group (**A**,**B**).

Fluorescence images were taken in TIF format using a fluorescence microscope (Leica DM 6000B, Leica Microsystems, Heerbrugg, Switzerland, Leica CTR 6000, Leica Microsystems, Heerbrugg, Switzerland) with the pertinent software (LAS AF 6000, Leica Microsystems, Heerbrugg, Switzerland). Special filters were used (green for calcein, and purple for tetracycline) and combined to an overlay image. Measurements of different areas were then performed using the free download software ImageJ 1.52a (https://imagej.nih.gov/ij/download.html) (accessed on 21 August 2020) (Figure 2 and Figure 3).

### 2.5. Study Variables & Statistic Analysis

The percentage of dentin growth area between the two fluorescence marks (tetracycline and calcein) was analyzed, with respect to the global area of the mineralized tooth excluding the area inside the calcein mark, depending on the different tested materials (NC, MTA, F1, or F2). The same values were analyzed in the two (A) and (B) cuts (Figure 2 and Figure 3).

The Kolmogorov–Smirnov normality test was carried out to examine if variables were normally distributed. Then, statistical analysis was carried out by one-way ANOVA test for parametric data. Statistical significance, indicative that the averages between the groups differ, was interpreted as *p* < 0.05. To determine the average difference between the compared groups, the Bonferroni test was used.

## 3. Results

Five samples were excluded, either due to traumatic injuries during the procedure or during the study time or because anatomical alterations (such as pulp size or total area of the tooth), with very different dimensions when compared with the rest of the group. Those were present in one animal and could be related to specific anatomical variations of each rabbit or fluctuation in wear and eruption during this period [15]. Consequently, 27 samples were submitted to analysis: 13 were maxillary incisors (48.1%) and 14 mandibular incisors (51.9%); the distribution of treatments (NC -negative controls, MTA -positive controls, PPGF -F1- and PRGF -F2-) in the analyzed samples is to be found in Table 1.

In all four groups, dentine formation was observed, shown as a circular pattern all around the root canal. The calcein mark is visible with the green filter closer to the canal, internal to the concentric tetracycline mark, visible thanks to the purple filter (Figure 2 and Figure 3). The two fluorescence marks (tetracycline and calcein) were observed in all but five of the samples, where only the tetracycline mark could be observed in the (A) section, due to the apposition of dentin pattern in apicocoronal direction; in those cases, in absence of calcein mark, the internal limits of the dentin growth area were measured from the dentine walls of the canal.

**Figure 4:**
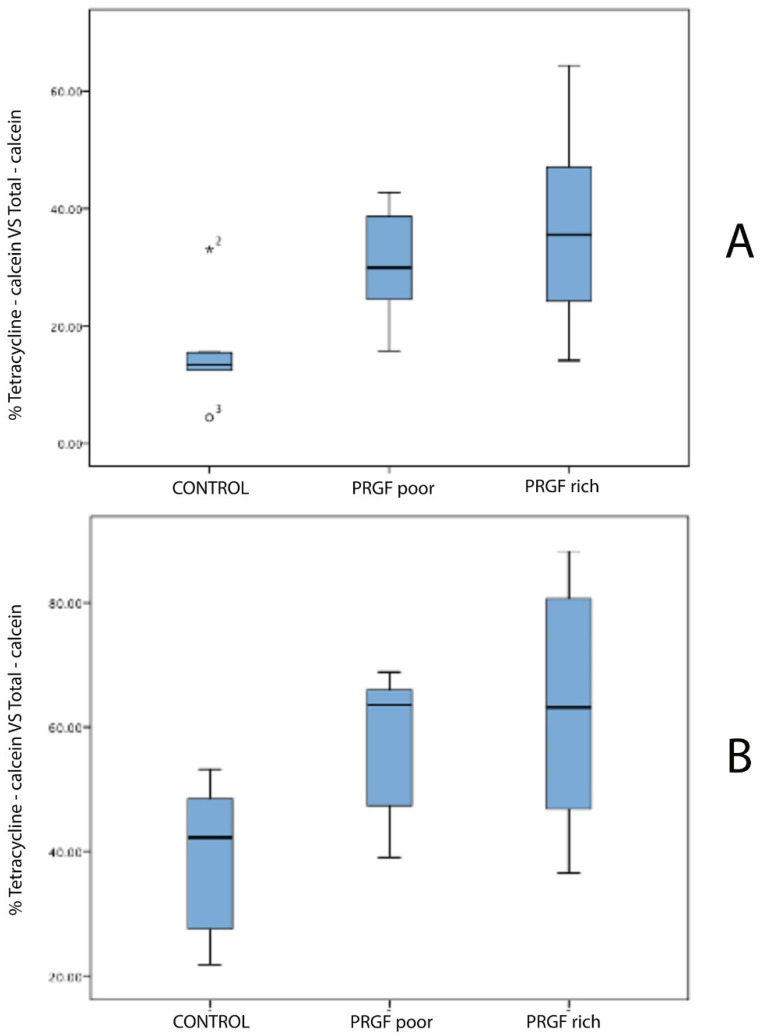
Bar graphs of median of the percentages for (**A**,**B**) sections with the different treatments.

The measurements were calculated for the two sections of the 22 teeth. The highest percentage of dentin growth was observed in the F2 group (PRGF), 63.25% in the (B) sections, and 36.52% in the (A) sections. The percentage of dentinogenesis observed in the F1 group (PPGF) was 57.63% in the (B) sections, and 30.12% in the (A) sections. For the MTA group (positive controls) the percentage of dentin growth was 38.64% in the (B) sections and 15.74% in the (A) sections. The group with lowest percentage of dentin growth was the NC group (negative controls): 29.22% in the (B) sections and 7.82% in the (A) sections (Table 1) (Figure 4).

One-way ANOVA test, Bonferroni test, and Kolmogorov–Smirnov normality test were performed with the following results:

For Sections A (parametric): One-way ANOVA test: *p* = 0.001; Bonferroni Test: Negative Control vs. Positive Control: *p* = 0.99, Negative Control vs. poor PRGF: *p* = 0.012, Negative Control vs. rich PRGF: *p* = 0.001, Positive Control vs. poor PRGF: *p* = 0.21, Positive Control vs. rich PRGF: *p* = 0.03, poor PRGF vs. rich PRGF: *p* = 0.99;

For Sections B (parametric): One-way ANOVA test: *p* = 0.001; Bonferroni Test: Negative Control vs. Positive Control: *p* = 0.99, Negative Control vs. poor PRGF: *p* = 0.007, Negative Control vs. rich PRGF: *p* = 0.001, Positive Control vs. poor PRGF: *p* = 0.13, Positive Control vs. rich PRGF: *p* = 0.03, poor PRGF vs. rich PRGF: *p* = 0.99;

The results of Kolmogorov–Smirnov normality test was: Sections A: Negative Control: *p* = 0.33; Positive Control: *p* = 0.31; poor PRGF: *p* = 0.15, rich PRGF: *p* = 0.13; Sections B: Negative Control: *p* = 0.21; Positive Control: *p* = 0.20; poor PRGF: *p* = 0.25, rich PRGF: *p* = 0.15.

There was no statistical difference in dentin growth area, neither in (A) nor in (B) sections, among F1 and F2, nor between MTA group and negative control or among F1 group and MTA group. However, statistically significant difference comparing dentin growth areas of NC group with F1 and F2 groups was observed. Furthermore, a significant difference was found between F2 group and MTA group, with *p* = 0.03 for (A) and (B) sections (Table 1).

## 4. Discussion

Numerous studies have described the use of rodents as a model to evaluate reactions to bioactive molecules with good results [16,17], both in form and function, histology, and physiology [18]. Usually, the studied teeth are molars or incisors, the latter having the advantage of a better access to perform the perforation and place the bioactive molecules onto the pulp tissue. Note that rodent’s incisors continuously grow; in this they are very different from human teeth [13]. Despite this fact, rodent’s incisors have also been validated as a study model, useful for the evaluation of potential human dental pulp response [19,20,21,22].

Moreover, the fluorochromes have been conveniently used to determine dentin growth rates in rabbit incisors [13,23]. The fluorescence microscopic markers present the advantages of not being an invasive procedure and not causing toxicity [24]. Wyss et al. (2016) showed that the determination of growth rates with fluorescent markers gives in rabbit teeth similar results as those obtained by other methods [13]. One of the limitations was the standardization of the teeth sections during histological preparation; for a longitudinal cut, the plane of the histological section has to be exactly parallel to the growth direction. It is very difficult to achieve in one sample, and impossible to reproduce in all samples. For this reason, in the present study, the cut was performed transversally to the tooth, obtaining a cross section of the incisor, with two concentric fluorescence marks (tetracycline and calcein) [23,24]. The areas measured between the marks can be compared with the total mineralized area of the tooth, in each section. In this way, the mistakes related to the cutting angle can be avoided.

The action of bioactive molecules in signaling the events of reparative and regenerative dentinogenesis has been extensively studied [25]. There are many growth factors, such as the BMP-2, BMP-4, BMP-7/OP-1, FGF-2, IGF-1 and -2, and TGF-b1, related with the induction of tertiary dentinogenesis and the process of odontoblastic differentiation from DPSCs [25,26]. A systematic review of Da Silva et al. (2016) concluded that the application of bioactive proteins enhanced tertiary dentin formation in direct and indirect pulp capping, with an initial lower inflammatory response, but no significant differences were found for pulpotomy [27].

Despite the advantages of growth factors, their use is still limited; because of their high cost, as well as the lack of understanding regarding some aspects, such as the correct signal, the adequate quantity and the containing biomaterials that should be used to obtain a specific response [25].

Autologous growth factors are a real alternative to recombinant growth factors, with fewer drawbacks for VPT. One possible source of growth factors for these treatments is the dentin itself; there are many growth factors (TGF-b, BMPs, FGF, platelet-derived growth factor (PDGF), VEGF and IGF) embedded in dentin extracellular matrix, during dentinogenesis. They can be released towards the pulp, under traumatic situations like caries, presence of root canal disinfectants and conditioning dentin tissue with EDTA. Those autologous dentin growth factors can then participate in cell homing of DPSCs to the wound site and induce tertiary dentinogenesis [28]. Some studies have shown greater levels of the TGF-β1 released from rabbit incisors than from human dentine, after EDTA conditioning [28,29]. This fact could be related with the increased reparative response of this animal model.

The other possible source of autologous growth factors is the PRGF, easily and rapidly obtained from the patient’s own blood. Once activated with CaCl_2_, PRGF results in a fibrin matrix with encapsulated platelets that secrete numerous growth factors (hepatocyte growth factor -HGF-, TGF-beta, PDGF, IGF, and VEGF) and drive tissue regeneration mechanisms in the local area [30]. Okada et al. (2016) showed that PDGF-αβ, PDGF-ββ, and VEGF concentrations in F1 were about 50% less than in F2, and that TGF-β concentration was markedly decreased in the supernatant [11]. That could be the reason why a lower dentin growth rate was found in F1 group than in F2 group. In previous in vivo and in vitro studies about regeneration of the dentine–pulp complex, it has been shown that PRGF and other platelet concentrates have the capacity to induce cell migration, pulplike tissue formation and differentiation of DPSCs into odontoblast-like cells [31].

Recently, the Spanish Agency of Medicines and Medical Devices (AEMPS) has classified the PRP as a medicinal product for human use but has clarified that the PRP preparation for autologous use does not imply an industrial processing, and thus it is not an industrially produced medicinal product. Furthermore, the AEMPS has indicated that PRP is not considered advanced-therapy medicinal products, according to the definition given by the European Union [Official Journal of the European Union (2007) EC/1394/2007 of the European Parliament and of the Council of 13 November 2007 on advanced therapy medicinal products and amending Directive 2001/83/EC and Regulation (EC) No 726/2004. (http://eurlex.europa.eu/LexUriServ/LexUriServ.do?uri=OJ:L:2007:324:0121:0137: en:PDF) (accessed on 14 April 2012)]. Currently, the autologous administration of PRP in the United States (U.S.) and Canada is considered as medical procedure and therefore is not subject to regulation by the U.S. Food and Drug Administration (FDA) and Health Canada.

Regarding the devises used to prepare the PRGF, they have FDA pre-market approval and also the CE mark as medical devices. Furthermore, the Endoret Kits used to prepare the PRGF have been included by the AEMPS in the list of closed systems.

In this study, the control group has been done with the application of MTA on the pulp exposure. Many investigators have reported that MTA was biocompatible and induced complete dentine bridge formation with no signs of inflammation [22]. However, there are very few studies that compare the results of platelet concentrates (PC) and MTA for VPT. These studies have not shown significant differences between the use of MTA or PC in pulpotomy procedures in permanent or temporary teeth, with a fair to poor evidence design of their human studies [32,33]. Orhan et al. (2012) showed in their animal model study, no statistically significant difference in reparative dentinogenesis amongst PC, Calcium hydroxide or MTA groups (*p* < 0.01), but they observed that the capping agents may influence the thickness of reparative dentin [22,33]. According to the results obtained in our study, the application of PRGF could serve an additional effect to place only MTA.

The model used in the present study contains a combination of three aspects that have not been under investigation before in VPT: the use of fluorescence marks to delimitate the dentin circumferential growth, the percentage of dentinogenesis with respect to the total mineralized area of the tooth and a comparison of incisal dentinogenesis (near the procedure site) with apical dentinogenesis.

In the present study, the teeth were embedded in plastic. Mineralized teeth are impossible to section when it is embedded in conventional media without previously demineralizing the tissue; this demineralization could consequentially provoke a poorer preservation of the mineralized structures [34]. That is why in the present study, the teeth were embedded in plastic. Nonetheless, plastic media limits the number of sections per tooth, for this reason it is necessary to select and standardize the cutting point.

Measuring only the area of dentin growth without taking into consideration the total mineralized area of the tooth could be a mistake. In fact, Müller et al. (2014) reported observations of fluctuating wear and eruption during long periods of consistent diets in rabbits [15]. Sometimes constancy of wear cannot be expected, and this is a concern that should be considered for the evaluation of the results. Excluding possible individual anatomical variations and comparing the dentin growth during the period of the study with the total area of the tooth in every rabbit could improve the significance of the sample avoiding different individual growth rates or between upper and lower incisors.

In the present study, after 28 days and for both incisal and apical sections, the higher percentage of dentin growth was observed in F2, followed by F1 and MTA. The lesser growth was found in the NC group. Anitua et al. (2013) showed that 30% of the growth factors contained in PRGF are released during the first hour; the other 70% during the next 8 days. They also mentioned the role of fibrin, capable of retaining almost 30% of the PRGF growth factors for more than one week [35]. Those results tend to establish that PC seem to have a limited action both in time and influence area, when used for VPT. The rabbit incisors, with a fast continuous growth (1.9 mm/wk for maxillary incisors and 2.2 mm/wk for mandibular incisors) [13], could present a limitation to evaluate the effect of PRGF in the dentinogenesis in a 28-day evaluation period; a shorter observation period could be indicated for this model.

Even though the three groups of study should be equal in size, in the present study, we had the limitation that MTA group and NC had less specimens than the F1 and F2 groups due to the difficulties of the study and in order to minimize the number of experimental animals following the guidelines for the care and use of experimental animals. These difficulties left a small sample size that could influence the outcome.

The results of our study should be viewed with caution due to the aforementioned limitations. It would be necessary further investigation with high-quality randomized controlled trials in a model more similar to the human one, or with less study time if done with rodents’ incisors to evaluate even better the effects of PC in.

Furthermore, there is a need to cost-effectiveness study for the use of PRGF in VPT.

## 5. Conclusions

The higher percentage of dentin growth was observed in the group where F2 had been applied as a pulp capping material, followed by F1 and MTA. The lesser growth was found in the NC group. Statistically significant difference (*p* < 0.05) has been found between the dentin growth areas F2 and MTA group, also statistically significant difference comparing dentin growth areas of NC group with F1 and F2 groups has been observed. It would be necessary further investigation to verify if this kind of animal model, with dental continuous growth, is appropriate to evaluate dentin growth, and also more research to determine which could be the optimal time period for this type of animal model.

## Figures and Tables

**Table 1:** Mean and median of the percentages for A and B sections.

	Section A		Section B	
	% Tetracycline-Calcein vs. Total-Calcein		% Tetracycline-Calcein vs. Total-Calcein	
	Mean (SD)	Median (Min-Max)	Mean (SD)	Median (Min-Max)
Negative Control (*n* = 5)	(7.82–3.29)	5.77 (5.03–11.88)	(29.22–2.32)	29.57 (25.79–32.23)
Positive Control (MTA) (*n* = 5)	(15.74–10.53)	13.41 (4.38–33.0)	(38.64–13.49)	42.21 (21.78–53.20)
Poor PRGF (*n* = 9)	(30.12–9.62)	29.98 (15.75–42.75)	(57.63–11.55)	63.56 (39.01–68.85)
Rich PRGF (*n* = 8)	(36.52–16.12)	35.54 (14.12–64.39)	(63.25–18.99)	63.15 (36.51–88.30)

## Data Availability

Data are contained within the article.
